# Improving synthetic CT accuracy by combining the benefits of multiple normalized preprocesses

**DOI:** 10.1002/acm2.14004

**Published:** 2023-04-24

**Authors:** Zheng Cao, Xiang Gao, Yankui Chang, Gongfa Liu, Yuanji Pei

**Affiliations:** ^1^ National Synchrotron Radiation Laboratory University of Science and Technology of China Hefei China; ^2^ Hematology & Oncology Department Hefei First People's Hospital Hefei China; ^3^ School of Nuclear Science and Technology University of Science and Technology of China Hefei China

**Keywords:** adaptive radiotherapy, CBCT, cycleGAN, normalization preprocessing method, synthetic CT

## Abstract

**Purpose:**

To investigate the effect of different normalization preprocesses in deep learning on the accuracy of different tissues in synthetic computed tomography (sCT) and to combine their advantages to improve the accuracy of all tissues.

**Methods:**

The cycle‐consistent adversarial network (CycleGAN) model was used to generate sCT images from megavolt cone‐beam CT (MVCBCT) images. In this study, 2639 head MVCBCT and CT image pairs from 203 patients were collected as a training set, and 249 image pairs from 29 patients were collected as a test set. We normalized the voxel values in images to 0 to 1 or −1 to 1, using two linear and five nonlinear normalization preprocessing methods to obtain seven data sets and compared the accuracy of different tissues in different sCT obtained from training these data. Finally, to combine the advantages of different normalization preprocessing methods, we obtained sCT_Blur by cropping, stitching, and smoothing (OpenCV's cv2.medianBlur, kernel size 5) each group of sCTs and evaluated its image quality and accuracy of OARs.

**Results:**

Different normalization preprocesses made sCT more accurate in different tissues. The proposed sCT_Blur took advantage of multiple normalization preprocessing methods, and all tissues are more accurate than the sCT obtained using a single conventional normalization method. Compared with other sCT images, the structural similarity of sCT_Blur versus CT was improved to 0.906 ± 0.019. The mean absolute errors of the CT numbers were reduced to 15.7 ± 4.1 HU, 23.2 ± 7.1 HU, 11.5 ± 4.1 HU, 212.8 ± 104.6 HU, 219.4 ± 35.1 HU, and 268.8 ± 88.8 HU for the oral cavity, parotid, spinal cord, cavity, mandible, and teeth, respectively.

**Conclusion:**

The proposed approach combined the advantages of several normalization preprocessing methods to improve the accuracy of all tissues in sCT images, which is promising for improving the accuracy of dose calculations based on CBCT images in adaptive radiotherapy.

## 1 INTRODUCTION

The radiotherapy treatment plan calculates the absorbed dose distribution in vivo based on anatomical and electron density information provided by the patients’ planning computed tomography (CT) images. Changes in anatomy during treatment may cause the actual absorbed dose in vivo to differ from the calculated value. These anatomical variations have led to the development of adaptive radiotherapy (ART),^1^, ^2^, ^3^, ^4^ in which plans are adjusted according to the actual clinical anatomical structures. Cone‐beam computed tomography (CBCT) images are widely used to monitor setup errors and anatomical variations. However, radiotherapy dose calculations based on CBCT images can be inaccurate due to scattering and other artifacts,^5^ which limits the applicability of CBCT images in ART. Recent studies have investigated converting CBCT images into synthetic CT (sCT) images with higher image quality through deep learning methods.^6^, ^7^, ^8^, ^9^ The sCT images have precise HU values similar to those of CT images and anatomical structures similar to those observed in CBCT images and may thus lead to accurate dose calculations in ART. Megavolt CBCT (MVCBCT) images have more noise, lower image contrast, and poorer image quality than kilovolt CBCT (kVCBCT) images. However, it is worth noting that the higher X‐ray energy greatly reduces the photon starvation and radiation hardening effects, making the streak and metal artifacts commonly seen in kVCBCT almost negligible.^10^ This has led many researchers to explore the feasibility of acquiring sCT images based on MVCBCT images.^10^, ^11^, ^12^, ^13^, ^14^

The deep learning processes use gradient descent, which requires data feature scaling before training. The CT numbers in different types of tissues vary greatly in CT images; the CT numbers in soft tissues are approximately 0 HU, and the CT numbers in cavities reach as low as −1000 HU, while the CT numbers in bony tissues can be greater than 1000 HU, and the CT numbers in teeth can reach 3000 HU. When training deep learning networks, different feature values should be treated as equally important; however, the numerical calculations are dominated by larger feature values, even if these are not the most important features. Thus, feature scaling is needed to normalize the different dimensional feature values to similar small ranges, such as 0 to 1 or −1 to 1.^15^, ^16^ On the other hand, larger differences in feature values result in large differences in gradient values, which requires a larger difference in the learning rate to guarantee model convergence. Feature scaling can accelerate model training by reducing the difference in the required learning rate.^15^, ^17^

Normalization is a common feature scaling method. Some research groups have used normalization methods in preprocessing before deep learning begins to generate sCT images based on CBCT images. The normalization range is typically 0 to 1 or −1 to 1, and the normalization methods are mainly linear normalization^11^, ^18^, ^19^, ^20^, ^21^, ^22^, ^23^ or nonlinear normalization approaches using the hyperbolic tangent function (Tanh).^24^, ^25^ CT images have unequal proportions of voxels for different tissues, with soft tissues being the most dominant (approximately more than seventy percent in the head). Before normalization, the voxel values in CT images are equal to the CT numbers, and the distribution of voxel values will be different after normalization by different methods in the preprocessing stage. The voxel values corresponding to soft tissues after linear normalization preprocessing (min‐max normalization) account for only approximately ten percent of the total voxel value range, which may not be effectively trained. In contrast, the nonlinear normalization preprocessing method can greatly increase the distribution range of voxel values corresponding to soft tissues after normalization and improve the training effect of soft tissues, but the cavities and bony tissues will become inaccurate again due to insufficient training. CT images are the basis for calculating the three‐dimensional dose distribution in patients, which is related to the efficacy and safety of radiotherapy, so any accuracy of body tissues should be guaranteed. However, the traditional single image normalization preprocessing method does not seem to ensure that all body tissues are adequately trained simultaneously. To the best of our knowledge, no studies have compared the impact of different normalization preprocessing methods on the quality of generated sCT images, which is explored in this study. In this study, the cycle‐consistent adversarial network (CycleGAN) model was used to generate sCT images based on MVCBCT images. We demonstrate that different image normalization preprocesses can lead to different accuracies in different CT number ranges for each set of sCT images obtained from training. We then select, crop, stitch, and smooth the above different sCT images to obtain a new set of images that combines the advantages of multiple normalization preprocessing methods to ensure the accuracy of all body tissues.

## 2 METHODS

### 2.1 Research process

**FIGURE 1:**
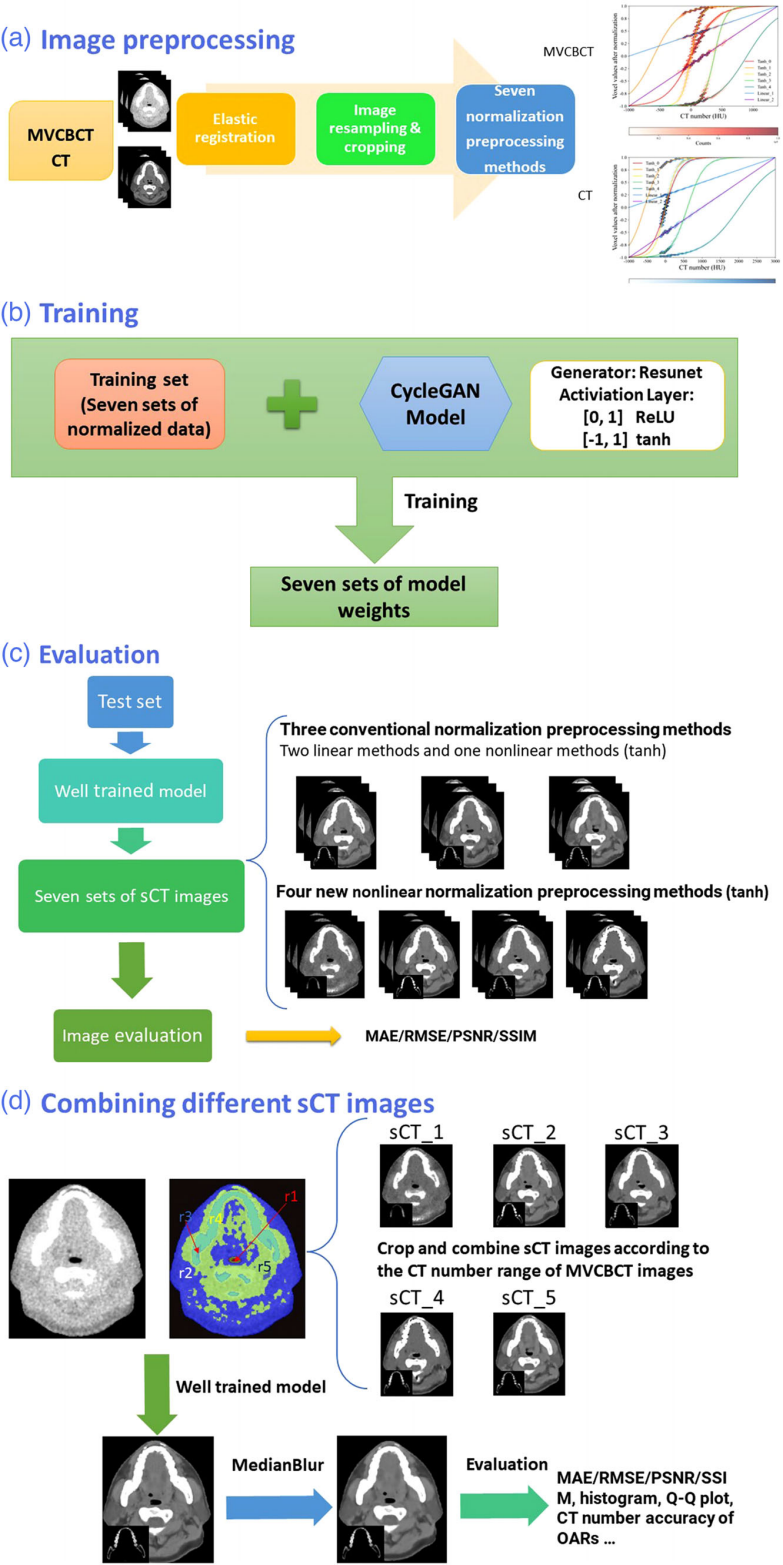
Schematic diagram. The process of this research can be divided into four parts: (a) image preprocessing, (b) training, (c) evaluation, and (d) combining different synthetic computed tomography (sCT) images.

As shown in Figure 1, the research procedure in this study can be divided into four steps. First (Figure 1a), the paired MVCBCT and CT images were preprocessed, including elastic registration, resampling, cropping, and normalization. Among the above preprocessing steps, normalized preprocessing is the most critical step and is also a crucial step in the entire study. We used different linear and nonlinear normalization preprocessing methods to normalize the voxel values of CT and MVCBCT images from the original CT number range to 0 to 1 or −1 to 1, respectively. Different normalization preprocessing methods change the distribution of voxel values of different body tissues after normalization. This affects the accuracy of different body tissues in sCT images. Second (Figure 1b), the CycleGAN model was trained by the above paired data normalized by seven different methods. Third (Figure 1c), the quality of the images generated by the seven trained models was evaluated on the testing cases. Finally, (Figure 1d), several data normalization preprocessing methods were combined to generate the final sCT images, and the image quality and CT numbers of organs at risk (OARs) were evaluated.

### 2.2 Data acquisition and preprocessing

In this study, paired CT and MVCBCT images were collected from 232 radiotherapy head patients. A total of 2639 image pairs from 203 patients were randomly selected as the training set, and the remaining 29 patients and 249 image pairs were used as the test set. The CT numbers of dental tissues are much larger than those of other bony tissues in the head, so including dental tissues in the images helps to investigate the effect of different normalization preprocessing methods on the accuracy of high CT number tissues in sCT images. Therefore, we selected only head CT images containing dental tissues for investigation in this study. The CT images were obtained with a Siemens SOMATOM Spirit helical CT scanner (tube voltage is 130 kV, slice thickness is 3 mm). Paired MVCBCT images were obtained in the first fraction of treatment (Siemens Artiste Medical Electron Linear Accelerator, 6 MV, 0.54 mm × 0.54 mm × 0.54 mm). The maximum CT number in the CT images did not exceed 3000 HU, and the maximum CT number in the MVCBCT images did not exceed 1400 HU. The MVCBCT images were elastically deformed according to the planning CT images to ensure that the spatial positions between the two images were as one‐to‐one as possible, and the deformed images were resampled to a spacing of 1 mm × 1 mm × 1 mm and cropped to 256 × 256 voxels.

### 2.3 Different normalization preprocessing methods

Various research groups have described linear normalization^11^, ^18^, ^19^, ^20^, ^21^, ^22^, ^23^ and nonlinear normalization preprocessing methods using the hyperbolic tangent function (Tanh),^24^, ^25^ where $\mathrm{Tanh}\left(x\right)\;=\;\frac{e^{x}-e^{-x}}{e^{x}+e^{-x}}$. With reference to the above literature,^11^, ^18^, ^19^, ^20^, ^21^, ^22^, ^23^, ^24^, ^25^ we defined two linear normalization and one nonlinear normalization preprocessing methods employed in this work as follows:

- $N_{img}=\left(HU_{img}-HU_{img,min}\right)/\left(HU_{img,max}-HU_{img,min}\right)\;$
- $N_{img}=2\times \left(HU_{img}-HU_{img,min}\right)/\left(HU_{img,max}-HU_{img,min}\right)\;-1$
- $N_{img}=\;tanh\left(\left(HU_{img}-a_{img}\right)/b_{img}\right),\;\;a_{CT}=a_{MVCBCT}\;=\;0,\;\;b_{CT}=b_{MVCBCT}\;=\;400$

where $N_{img}$ represents the normalized voxel value and $HU_{img}$ represents the CT number of a voxel in the image. The subscript *img* indicates the image type, corresponding to the CT or MVCBCT images.

In the image set used in the paper, values between −400 and 150 HU (soft tissues) only make up 13.75% of the total voxel value range, but the corresponding voxels account for approximately 75.55% (Table 1) of the total voxels. After two linear normalizations (I, II), values between −400 and 150 HU are mapped to 0.15 to 0.29 and −0.70 to −0.43, respectively (Table 2), with a ratio of values still 13.75% (Table 3), which may result in 75.55% of the images (soft tissue) not being effectively trained. The nonlinear normalization preprocessing method III increased the proportion of soft tissue voxel values to 56% (Table 3). Thus, the soft tissue portions of the images can be more thoroughly trained, leading to improved accuracy. However, the proportion of the 1500–3000 HU mapping interval decreased from 37.5% to 0.06% (Table 3) after normalization with method III, resulting in images of high CT number tissues (e.g., dental tissue) not being effectively trained. In radiotherapy, CT images are used to accurately calculate dose distributions, impacting the efficacy and safety of the treatment. Improving the voxel value distribution after normalization preprocessing for different tissues separately by changing the normalization preprocessing method for better training is feasible.

**TABLE 1:** Ratio of voxels in different CT number ranges in CT images

CT number ranges (HU)	[−1000, −400]	[−400, 150]	[150, 1500]	[1500, 3000]
Ratio of voxels (%)	0.95	75.55	21.44	2.06

*Note*: The number of voxel points corresponding to soft tissue with CT numbers in the range of −400 to 150 HU accounted for the majority of the CT images used in this study.

Abbreviation: CT, computed tomography.

**TABLE 2:** Voxel values corresponding to different CT number after various normalization preprocessing of CT images

CT number (HU)	−1000	−400	150	1500	3000
Method I	0.00	0.15	0.29	0.63	1.00
Method II	−1.00	−0.70	−0.43	0.25	1.00
Method III	−0.98	−0.76	0.36	1.00	1.00
Method IV	−0.76	0.39	0.94	1.00	1.00
Method V	−1.00	−0.87	0.46	1.00	1.00
Method VI	−1.00	−0.98	−0.76	0.96	1.00
Method VII	−1.00	−0.99	−0.96	0.49	0.76

*Note*: Voxel values corresponding to different CT number after preprocessing with different normalization methods are very different.

Abbreviation: CT, computed tomography.

**TABLE 3:** Proportion of voxel values (%) corresponding to different CT number ranges after different normalization preprocessing

CT number ranges (HU)	[−1000, −400]	[−400, 150]	[150, 1500]	[1500, 3000]
Method I	15.00	13.75	33.75	37.5
Method II	15.00	13.75	33.75	37.5
Method III	11.25	56.00	32.03	0.06
Method IV	57.58	27.31	3.19	0.01
Method V	6.37	66.61	26.89	<0.01
Method VI	1.08	10.76	86.28	1.80
Method VII	0.49	1.40	23.32	62.67

*Note*: Changing the image normalization preprocessing method will change the body tissues in which the normalized voxel values will occupy the main distribution.

Abbreviation: CT, computed tomography.

We developed four other normalization preprocessing methods by changing the scaling center ($a_{img}$) and proportion ($b_{img}$) in method III. These methods ensure that the corresponding voxel values of different tissues (cavities, soft tissues, bony tissues (excluding teeth), dental tissues) fit the main distribution in the range from −1 to 1 after image normalization preprocessing. These four normalization preprocessing methods can be expressed as IV to VII:

IV $N_{img}=\;tanh\left(\left(HU_{img}-a_{img}\right)/b_{img}\right),\;a_{CT}=-575,\;\;a_{MVCBCT}=-600,\;\;b_{CT}=\;425,\;\;b_{MVCBCT}=\;400$
V $N_{img}=\;tanh\left(\left(HU_{img}-a_{img}\right)/b_{img}\right),\;a_{CT}=a_{MVCBCT}\;=\;0,\;\;b_{CT}=\;300,\;\;b_{MVCBCT}=\;150$
VI $N_{img}=\;tanh\left(\left(HU_{img}-a_{img}\right)/b_{img}\right),\;a_{CT}=\;600,\;\;a_{MVCBCT}=\;375,\;\;b_{CT}=\;450,\;\;b_{MVCBCT}=\;175$
VII $N_{img}=\;tanh\left(\left(HU_{img}-a_{img}\right)/b_{img}\right),\;a_{CT}=\;2025,\;\;a_{MVCBCT}=\;900,\;\;b_{CT}=\;975,\;\;b_{MVCBCT}=\;500$

Table 2 shows the changes in voxel values of CT images for different normalization preprocessing methods (−1000 HU, −150 HU, 400 HU and 3000 HU). Methods IV to VII modify the voxel values after normalization preprocessing by changing the values of $a_{img}$ and $b_{img}$ in III, altering the range of voxel values for different tissues. As shown in Table 3, after normalized preprocessing of IV to VII, the widest distribution of voxel values is obtained for cavities (−1000 to −150 HU), soft tissue (−150 to 400 HU), bony tissue (excluding teeth, 400 to 1500 HU), and dental tissue (1500 to 3000 HU), respectively, in the CT images.

The synthetic images generated by the CycleGAN models after normalizing the CT and MVCBCT images according to the above seven normalization methods in the preprocessing are named Linear_1, Linear_2, Tanh_0, Tanh_1, Tanh_2, Tanh_3 and Tanh_4. These images as a whole are referred to as “uncombined sCT images” in later sections.

### 2.4 Structure and training of the CycleGAN model

The CycleGAN model is an unsupervised network that has been widely used to generate synthetic CT images.^5^, ^7^, ^10^, ^26^, ^27^, ^28^ The CycleGAN architecture is consistent with that of the original paper described by Zhu et al. (2017), which includes two generators (Gmvcbct‐ct and Gct‐mvcbct) and two discriminators (Dct and Dmvcbct), using ResUNet by Xiao et al. (2018) and 70×70 PatchGAN described by Zhu et al. (2017) as generators and discriminators, respectively.^29^, ^30^ Gmvcbct‐ct converts MVCBCT to CT images and Gct‐mvcbct converts CT to MVCBCT images. Dct is used to discriminate sCT from real CT images and Dmvcbct is used to discriminate sMVCBCT from real MVCBCT images. In training, Gct‐mvcbct also converts the sCT images generated by Gmvcbct‐ct into cycle MVCBCT images and ensures that cycle MVCBCT is as consistent as possible with real MVCBCT images. The activation functions in the last ResUNet layer are ReLU and tanh when the normalization range is 0 to 1 and −1 to 1, respectively. The Python deep learning library PyTorch was employed as a backend to achieve the CycleGAN architecture. An Nvidia Quadro RTX 6000 GPU with 24 G memory was used to train the model. The initial learning rate was set to 0.002, the number of epochs was set to 200, and the learning rate decreased linearly from 0.002 to 0 in the last 100 epochs.

### 2.5 Cropping and combining the sCT images

As discussed in the “Different normalization preprocessing methods” section, different normalization preprocessing methods can alter the distribution of voxel values for different tissues. The more widely distributed the voxel values are, the better the data of the body tissues will be trained, and the more accurate their synthetic images will be. Therefore, the sCT images obtained by different normalization preprocessing methods will have higher accuracy in different body tissues. We chose MVCBCT images (within the skin) as a benchmark for segmentation of image regions, as the sCT images in this study were all generated based on MVCBCT images with consistent anatomical structures. We select the most accurate sCT images in different image regions compared to the planning CT images. To verify the generalization ability of the model, the cropping and stitching of the sCT images in the validation set are based on the selection result of the training set. For example, as shown in Figure 1(d), assume the training set MVCBCT images (the part inside the skin) are divided into five image regions (r1–r5) according to the CT number from the smallest to the largest, and the most accurate sCT images in these five regions are sCT_1 to sCT_5. Then, in the validation set, the MVCBCT images are segmented into five regions according to the same criteria, and the r1 part of sCT_1, r2 part of sCT_2, r3 part of sCT_3, r4 part of sCT_4, and r5 part of sCT_5 are each cropped and reassembled into one image. The final combined images are expected to be more accurate than all prior sCT images. In this study, the MVCBCT images are first smoothed with an average filter (OpenCV's cv2.blur, kernel size 5) and then segmented into 48 regions by CT number in 50 HU units. The structural similarity index (SSIM), which is typically used to compare the similarity of two images, was chosen to select the most accurate sCT images in each of the 48 regions in the training set. Based on the selection results in the training set, we cropped the corresponding sCT images in the test set from each of the 48 regions and stitched them together to create the combined image—sCT_Combine. We obtained sCT_Blur by filtering sCT_Combine with a median filter (OpenCV's cv2.medianBlur, kernel size 5) because we found that the stitching edges of the different sCT images in the sCT_Combine images were less natural and smooth to the human eye.

### 2.6 Image accuracy evaluation

The relative electron density (RED) is one of the main bases for radiotherapy dose calculation.^12^ To compare the differences in the RED distributions due to noise and artifacts between the MVCBCT and CT images, the MVCBCT images were converted to CT images using CT number‐RED correction curves. The original MVCBCT images are denoted as MVCBCT_1, and the converted images are denoted as MVCBCT_2. We evaluated the accuracy of the sCT images compared to planning CT images qualitatively by eye and quantitatively using metrics such as the mean absolute error (MAE), root mean square error (RMSE), peak signal‐to‐noise ratio (PSNR) and structural similarity index (SSIM) as described by Chen et al.^19^ Lower MAE and RMSE and higher PSNR and SSIM indicate better image quality.

The MAE is used to calculate the average of the absolute error of all voxel values between the two CT images.

(1) $$MAE\;\left(I_{1},I_{2}\right)=\frac{1}{n}\;\sum_{i\;=\;1}^{n}\left|I_{1,i}-I_{2,i}\right|\;$$

The RMSE measures the deviation between the model‐generated sCT images and the planning CT images and is more sensitive to outliers in the deviation of voxel values.

(2) $$RMSE\left(I_{1},I_{2}\right)=\sqrt{\frac{1}{n}\sum_{i=1}^{n}{\left(I_{1,i}-I_{2,i}\right)}^{2}}\;\;$$

PSNR is the most common and widely used objective measure of image distortion.

(3) $$PSNR\left(I_{1},I_{2}\right)=\;10\times \log_{10}\left(\frac{MAX^{2}}{RMSE{\left(I_{1},I_{2}\right)}^{2}}\right)$$

SSIM mainly considers three key features of an image: brightness, contrast and structure, and can measure the similarity of two images. SSIM is a perceptual model that is more in line with the intuition of the human eye. It is different from MSE, RMSE, and PSNR, which measure the absolute error between images.

(4) $$SSIM\left(I_{1},I_{2}\right)=\frac{\left(2\mu_{I_{1}}\mu_{I_{2}}+c_{1}\right)\left(2\sigma_{I_{1},I_{2}}+c_{2}\right)}{\left(\mu_{I_{1}}^{2}+\mu_{I_{2}}^{2}+c_{1}\right)\left(\sigma_{I_{1}}^{2}+\sigma_{I_{2}}^{2}+c_{2}\right)}\;$$

In Equations (1) to (4), MAX is the maximum CT number in the image. *I*_1_ and *I*_2_ represent the two images to be compared. $I_{1,i}$ and $I_{2,i}$ refer to the HU values of the i‐th voxel in images *I*_1_ and *I*_2_. $\mu_{I_{1}}$ and $\mu_{I_{2}}$ represent the mean of the voxel values in *I*_1_ and *I*_2_, respectively. $\sigma_{I_{1}}$ and $\sigma_{I_{2}}$ represent the standard deviation of the voxel values in *I*_1_ and *I*_2_, respectively. $\sigma_{I_{1},I_{2}}$ represents the covariance of the voxel values in *I*_1_ and *I*_2_. *c*_1_ and *c*_2_ are constants.

In the study by Vinas et al. (2021), images with CT numbers within the range of [−400, 150] HU were defined as soft tissue areas, and images with CT numbers larger than 150 HU were defined as bony tissue areas.^10^ The same division standard is adopted in this study, and regions with [−1000, −400] HU are defined as cavities. The voxels outside the skin are excluded when evaluating the overall image. When images of different tissues were evaluated, only the voxels with CT numbers within a certain range were assessed. We used integrated two‐dimensional histograms and quantile‐quantile (Q‐Q) plots to analyze how similar the CT number distributions of different sCT images were to the planning CT images (Figure 5). The more accurate the CT number of the sCT image, the brighter and more concentrated the hexagon around the 45‐degree line. The closer the Q‐Q plot is to a 45‐degree straight line, the more similar the CT number distribution is between the two sets of CT images. Two associate chief physicians of radiotherapy outlined and reviewed the contours of the major OARs (oral cavity, parotid, spinal cord, cavity, mandible, and dental tissue) on the planning CT images and reverified the accuracy of the contour reproduction after we copied the contours onto each set of sCT images. These contours were used to evaluate the difference between the CT numbers of the corresponding voxel points in different images. Wilcoxon signed‐rank tests for two related samples were performed using IBM SPSS Statistics software^31^ to analyze whether the differences between the accuracies of the various sCT images were statistically significant.

## 3 RESULTS

The following sCT images had the highest SSIM values in different CT number ranges in the MVCBCT_1 images: [−350, −100] HU and [200, 400] HU, Tanh_0; [−100, −50] HU and [−800, −350] HU, Tanh_1; [−1000, −900] HU and [−50, 200] HU, Tanh_2; [−900, −800] HU and [400, 450] HU, Tanh_3; and [450, 1400] HU, Tanh_4. The sCT_Combine image was composed of sCT images obtained within the above ranges.

**FIGURE 2:**
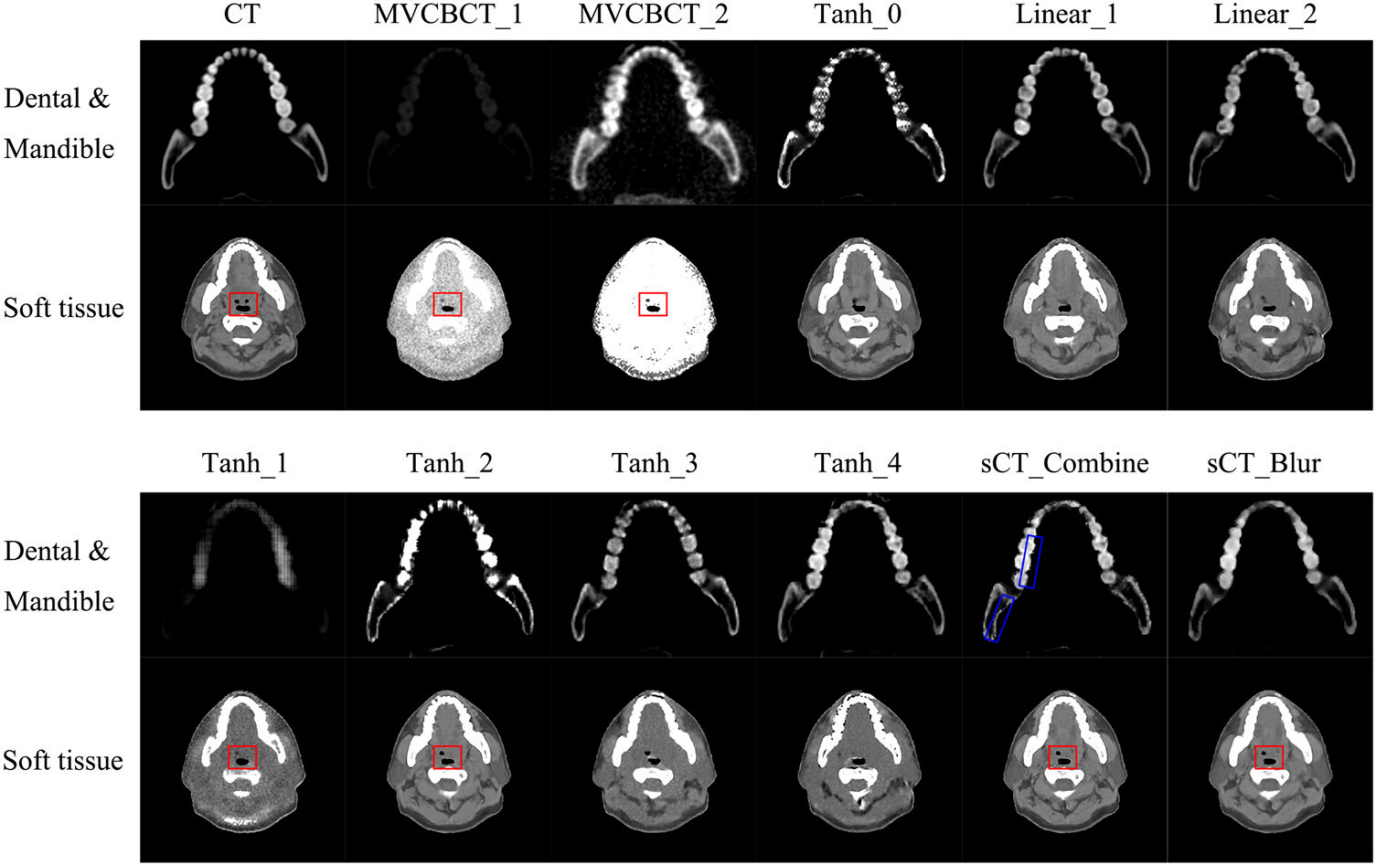
Comparison of the CT, MVCBCT, and sCT images. The display windows are [−260, 340] HU for soft tissue and [500, 2500] HU for dental tissue and the mandible. Different sCT images have their own advantages and disadvantages in various tissue regions, while both soft and bony tissues in sCT_Blur are close to the planning CT images. CT, computed tomography; MVCBCT, megavolt cone‐beam CT; sCT, synthetic computed tomography.

Figure 2 compares the sCT, MVCBCT, and planning CT images, with similarity determined qualitatively by eye. Although MVCBCT_2 is electron density corrected, its image, similar to MVCBCT_1, has a very different appearance from the CT image. The cavity shapes of Tanh_1 and Tanh_2 in the uncombined sCT images look more consistent with the MVCBCT images. sCT_Combine and sCT_Blur inherit this advantage (as shown by the red boxed line in Figure 2). The teeth and mandibles in Tanh_4, Linear_1, and Linear_2 look closer to the planning CT. The teeth in Tanh_3 are darker compared to the planning CT, but the mandible images look accurate. The soft tissue structures in the Tanh_0, Tanh_2, Linear_1, and Linear_2 images appear finer than those in the other sCT images and are consistent with the planning CT. sCT_Combine images perform well for both soft and bony tissues, but appear less smooth at the edges of some anatomical structures (such as the edges of the teeth and mandible in the blue line box in Figure 2) than the sCT_Blur images processed with the median filter. Figures S1 and S2 demonstrate the generalizability of the model in this work. All sCT_Blur images appear to be close to the planning CT images, both for soft tissue (Figure S1) and bony tissue (Figure S2). Additionally, the anatomical structures in the sCT_Blur images are consistent with MVCBCT_1 (shown in the red boxed line in the figure).

**FIGURE 3:**
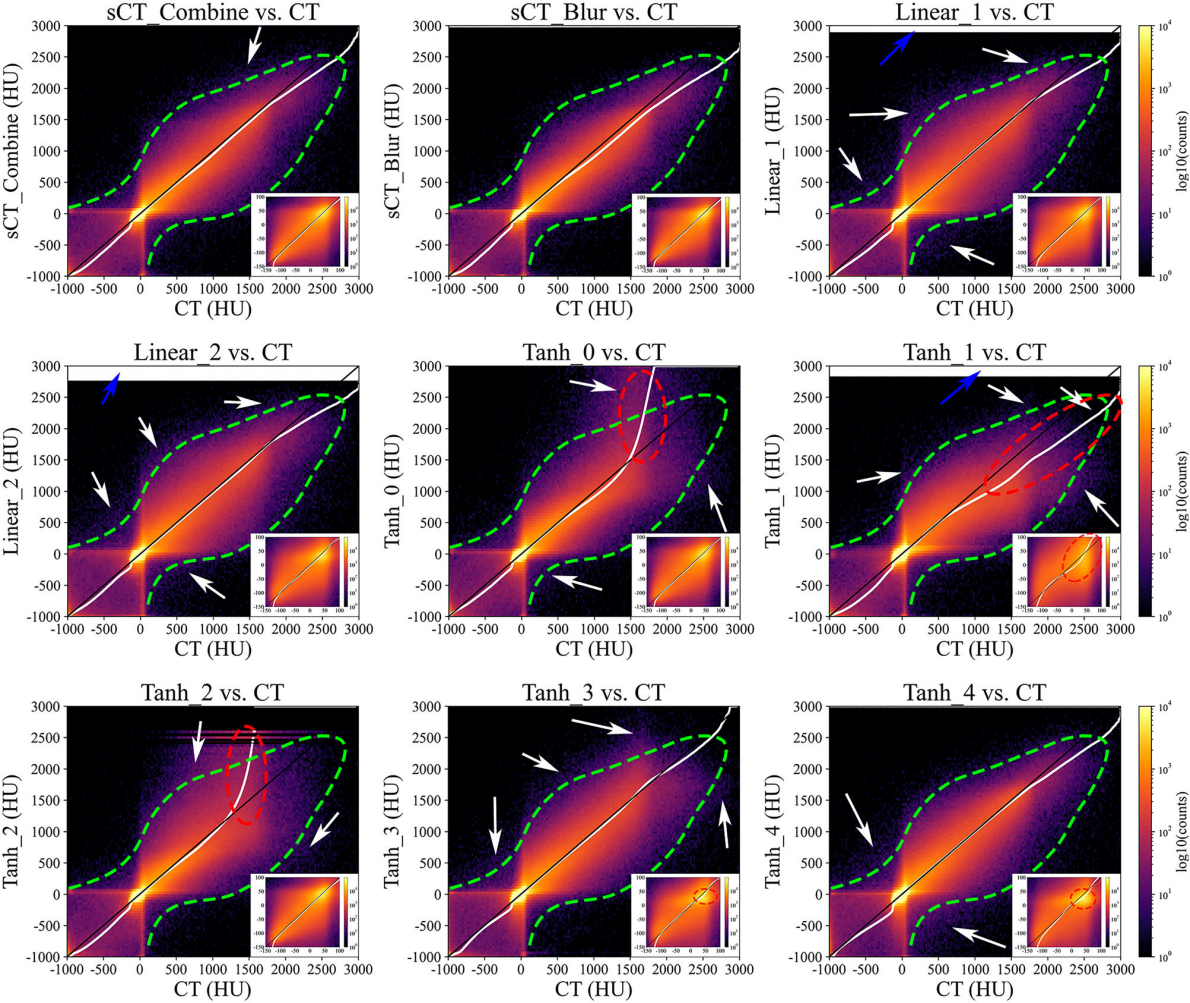
Two‐dimensional histograms and Q‐Q plots of the sCT_Combine, sCT_Blur and uncombined sCT images relative to the planning CT images in the range of [−1000, 3000] HU. The brighter the color near the 45‐degree line and the more concentrated the bright color block, the more accurate the CT number of the sCT image and the smaller the dispersion of the CT number error. The white curve is the Q‐Q plot. If the Q‐Q plot deviates too much from the 45‐degree line, it indicates that the CT number distribution of the two images has a large difference. To better compare the soft tissue regions of the images, 2D histograms were redrawn and magnified in the lower right corner of each subplot in the range of −150 to 100 HU. The green dashed lines in the subfigures indicates the main concentration area of brighter blocks in sCT‐Blur. CT, computed tomography; sCT, synthetic computed tomography.

Figure 3 shows the consistency of the CT number distribution of sCT_Combine, sCT_Blur and each group of uncombined sCT images with the planning CT images. Each group of uncombined sCT images had different accuracies in different CT number intervals, consistent with Figure 2. The soft tissue in Tanh_0 and Tanh_2 is relatively accurate, but the bony tissue (CT number > 400 HU) differs from the planning CT images (shown by the red ellipse). Both soft tissue and bony tissue in Tanh_1 are inaccurate (shown by the red ellipse). Tanh_3 and Tanh_4 are relatively inaccurate in the soft tissue (shown by the red ellipse) but accurate in the bony tissue. Linear_1 and Linear_2 are accurate in both soft and bony tissues, but the highest CT numbers are obviously lower than the planning CT (white area shown by the blue arrow). sCT_Combine images are accurate in both soft and bony tissues. In addition, the brighter color blocks in sCT_Combine versus CT are basically concentrated within the green dashed line compared to the uncombined CT images (shown by the white arrow), indicating that the dispersion of CT number errors in sCT_Combine images is lower, while the dispersion of CT number errors in sCT_Blur images is even lower after smoothing (shown by the white arrow in sCT_Combine vs. CT).

It is necessary to quantitatively compare the accuracy of each group of sCT images in different CT number intervals. Tables 4 and 5 show the accuracy of uncombined sCT images in different CT number intervals and the image quality improvement of sCT_Blur relative to uncombined sCT images quantitatively, where all sCT images are compared to planning CT images by metrics including MAE, RMSE, PSNR, and SSIM. The quantitative analysis shows the image quality variability of different uncombined sCT images in different CT number intervals and no single uncombined sCT image provided the best accuracy for all tissues. Among the uncombined sCT images, Tanh_2 and Tanh_4 are the most accurate images in the range of −400 to 150 HU (soft tissue) and 150 to 3000 HU (bony tissue), respectively. Tanh_1 has the best MAE, RMSE and PSNR metrics in the range of −1000 to −400 HU (cavity), while Tanh_0 has the highest SSIM in the same range. The sCT_Combine obtained by cropping and stitching the images from Tanh_0 to Tanh_4 combines their respective advantages and is more accurate than all uncombined sCT images within the entire skin ([−1000, 3000] HU). All image quality metrics of sCT_Blur are better than those of sCT_Combine (MAE = 69.5 ± 12.3 HU, RMSE = 140.5 ± 31.4 HU, PSNR = 29.3 ± 1.7 dB, SSIM = 0.906 ± 0.019).

**TABLE 4:** Evaluation of the different sCT and MVCBCT images within the different CT number ranges

Tissue	CT name	MAE (HU)	RMSE (HU)	PSNR (dB)	SSIM
Cavity [−1000, −400] HU	MVCBCT_1	410.8±112.8	441.6±114.9	19.4±2.2	0.349±0.130
	MVCBCT_2	255.4±143.8	306.9±171.6	23.3±4.0	0.530±0.153
	Linear_1	265.9±118.8	353.7±128.9	21.6±3.1	0.443±0.138
	Linear_2	272.0±121.1	364.5±136.5	21.4±3.2	0.463±0.123
	Tanh_0	221.4±101.9	298.9±113.7	23.1±3.0	0.517±0.141^a^
	Tanh_1	195.5±101.3^[Link]^, ^b^	268.9±117.6^[Link]^, ^b^	24.1±3.4^[Link]^, ^b^	0.515±0.164
	Tanh_2	220.8±98.6	298.9±111.9	23.1±2.9	0.494±0.136
	Tanh_3	294.4±127.4	383.2±127.1	20.8±2.8	0.384±0.130
	Tanh_4	308.8±125.6	383.7±126.8	20.8±2.8	0.353±0.121
	sCT_Combine	213.7±101.4	294.0±113.0	23.2±3.1	0.510±0.148
	sCT_Blur	212.8±104.6	291.4±115.4	23.4±3.2	0.529±0.145^b^
Soft tissue [−400, 150] HU	MVCBCT_1	96.4±49.6	112.9±47.6	31.7±3.5	0.926±0.013
	MVCBCT_2	205.1±136.1	258.8±142.5	25.0±4.5	0.720±0.098
	Linear_1	46.7±7.8	96.9±22.4	32.5±1.9	0.952±0.011
	Linear_2	45.4±6.8	92.2±21.4	33.0±2.0	0.955±0.010
	Tanh_0	39.4±6.1	76.6±20.7	34.6±2.1	0.962±0.009
	Tanh_1	54.2±8.4	91.2±21.2	33.0±1.8	0.925±0.022
	Tanh_2	37.3±6.1^a^	75.6±21.4^a^	34.8±2.1^a^	0.965±0.010^a^
	Tanh_3	44.5±6.0	85.2±18.1	33.6±1.7	0.958±0.008
	Tanh_4	48.9±6.6	95.2±18.5	32.6±1.6	0.951±0.009
	sCT_Combine	37.2±5.9	73.8±20.2^b^	35.0±2.1^b^	0.964±0.010
	sCT_Blur	36.1±5.9^b^	74.2±19.2	34.9±2.0	0.968±0.009^b^
Bony tissue [150, 3000] HU	MVCBCT_1	415.2±92.7	537.8±112.8	17.6±1.8	0.789±0.038
	MVCBCT_2	387.8±176.3	449.5±178.0	19.6±3.3	0.854±0.042
	Linear_1	253.9±42.7	341.7±64.6	21.5±1.3	0.834±0.036
	Linear_2	256.6±41.1	346.2±64.6	21.4±1.4	0.831±0.038
	Tanh_0	286.4±55.1	431.3±86.2	19.5±1.6	0.819±0.032
	Tanh_1	294.8±62.8	382.8±91.2	20.6±1.8	0.816±0.042
	Tanh_2	383.1±82.7	587.4±117.8	16.8±1.7	0.781±0.039
	Tanh_3	244.7±44.6	329.4±69.3	21.8±1.5	0.847±0.029
	Tanh_4	230.0±44.5^a^	313.1±73.7^a^	22.3±1.7^a^	0.856±0.029^a^
	sCT_Combine	215.1±43.5	293.5±72.1	22.9±1.6	0.864±0.030
	sCT_Blur	203.7±44.4^b^	275.1±73.6^b^	23.5±1.8^b^	0.880±0.029^b^

Abbreviations: CT, computed tomography; MAE, mean absolute error; MVCBCT, megavolt cone‐beam CT; PSNR, peak signal‐to‐noise ratio; RMSE, root mean square error; SSIM, structural similarity index.

^a^ The images with the best image quality index within the corresponding CT number range among the uncombined sCT images.

^b^ The images with the best image quality index within the corresponding CT number range among all the sCT images. No single uncombined sCT image provides the best accuracy for all tissues, and all tissues in sCT_Blur, which combines the advantages of individual uncombined sCT images, are the most accurate of the groups of sCT images.

**TABLE 5:** Evaluation of the different sCT images within the CT number range of [−1000, 3000] HU

CT name	MAE (HU)	RMSE (HU)	PSNR (dB)	SSIM
MVCBCT_1	160.8±34.5	262.9±41.1	23.8±1.4	0.808±0.020
MVCBCT_2	239.7±140.2	306.1±144.0	23.2±3.9	0.687±0.084
Linear_1	87.9±14.6	175.7±30.1	27.3±1.4	0.871±0.025
Linear_2	87.6±15.0	175.9±31.4	27.3±1.5	0.872±0.026
Tanh_0	86.7±12.2	199.1±27.2	26.1±1.1	0.886±0.021^a^
Tanh_1	101.1±16.5	188.1±39.1	26.7±1.6	0.845±0.021
Tanh_2	102.1±14.1	260.9±35.0	23.8±1.1	0.876±0.021
Tanh_3	84.6±13.6^a^	166.7±29.4	27.7±1.4	0.882±0.020
Tanh_4	85.7±14.4	165.5±31.6^a^	27.8±1.5^a^	0.876±0.020
sCT_Combine	72.5±12.6	147.0±31.4	28.9±1.6	0.897±0.020
sCT_Blur	69.5±12.3^b^	140.5±31.4^b^	29.3±1.7^b^	0.906±0.019^b^

Abbreviations: CT, computed tomography; MAE, mean absolute error; PSNR, peak signal‐to‐noise ratio; RMSE, root mean square error; sCT, synthetic computed tomography; SSIM, structural similarity index.

^a^ Images with the best MAE, RMSE, PSNR, and SSIM values among the uncombined sCT images.

^b^ The images with the best MAE/RMSE/PSNR/SSIM value among all sCT images. The sCT_Blur images have the best image quality of all sCT images.

**FIGURE 4:**
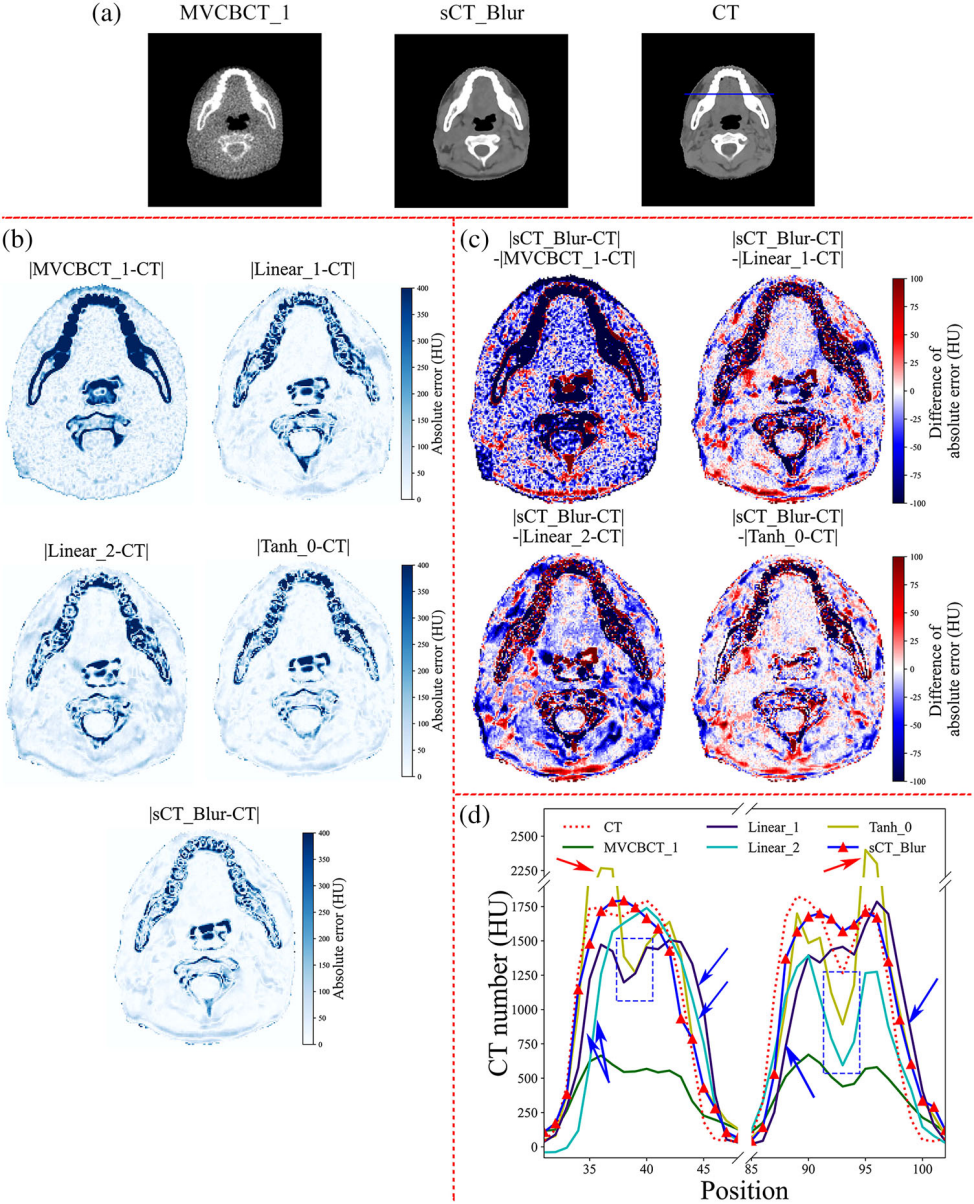
CT number accuracy comparison between sCT_Blur and MVCBCT_1, Linear_1, Linear_2, Tanh_0 images with planning CT image as reference image. (a) MVCBCT_1, sCT_Blur and planning CT image with a display window of [−260, 340] HU. (b) Absolute error maps of CT numbers of MVCBCT_1, Linear_1, Linear_2, Tanh_0, and sCT_Blur images with the planning CT image. The “|” in the title of the figure is the absolute value symbol. For example, |sCT_Blur‐CT| represents the absolute error map of the CT number of the sCT_Blur image and the planning CT image. The darker blue in the graph represents greater absolute CT number error from the planning CT image. (c) Difference maps of the absolute error of the CT numbers |sCT_Blur‐CT| versus |MVCBCT_1‐CT|, |Linear_1‐CT|, |Linear_2‐CT|, |Tanh_0‐CT|. Using |sCT_Blur‐CT|‐|Tanh_0‐CT| as an example, if the voxel point in the figure is blue, the CT value of sCT_Blur at that point is more accurate than Tanh_0. The darker the blue color, the more accurate the sCT_Blur image is relative to Tanh_0. The opposite is true for red. (d) CT number profiles of the planning CT, MVCBCT_1, Linear_1, Linear_2, Tanh_0, and sCT_Blur on the teeth where the blue line passes in (a). The sCT_Blur images show better accuracy than the Linear_1, Linear_2 and Tanh_0 images. CT, computed tomography; MVCBCT, megavolt cone‐beam CT; sCT, synthetic computed tomography.

While Tables 4 and 5 show that sCT_Blur image quality metrics are better compared to other sCT images (Linear_1, Linear_2, and Tanh_0) obtained by training with the CycleGAN model after preprocessing the images with three common normalization methods (methods I to III), Figure 4 provides a more visual comparison of image accuracy. sCT_Blur preserves the anatomical structure information of MVCBCT_1, while the image is closer to the planning CT image (Figure 4a). The absolute CT number error maps in Figure 4(b) compares the CT number accuracy of MVCBCT_1, sCT_Blur, and other sCT images (Linear_1, Linear_2, and Tanh_0) with the planning CT as the reference image. The result shows that all sCT images are more accurate than MVCBCT_1. In the dental tissue, the blue color in the |sCT_Blur‐CT| image is lighter relative to the absolute error maps of CT numbers in the other sCT images, representing a more accurate distribution of CT numbers in the dental tissue. We created Figure 4(c) to more directly compare the CT number accuracy of sCT_Blur with the other three sCT images. The darker blue color of a point in a map means that the absolute error of the CT number of sCT_Blur at that point is relatively smaller and sCT_Blur is more accurate. The opposite is true for red, and white means that the absolute error of the CT number of both images at that point is equal. In the two difference maps “|sCT_Blur‐CT|‐|Linear_1‐CT|” and “|sCT_Blur‐CT|‐|Linear_2‐CT|”, red and blue are mixed throughout the map, but overall, there are more blue points in the maps. This indicates that sCT_Blur is more accurate than Linear_1 and Linear_2. In map |sCT_Blur‐CT|‐|Tanh_0‐CT|, it is hard to tell by eye which color is more in the soft tissues, but the blue color is clearly more and darker in the dental part. This shows that the accuracy of the dental tissue is improved in sCT_Blur compared to Tanh_0. Figure 4(d) shows the CT number profiles for the portion of the tooth that passes through the blue line in Figure 4(a). Some of the teeth in Tanh_0 obviously have higher CT numbers than planning CT (shown by the red arrows). All three uncombined sCT images have some voxels in the teeth with much lower CT numbers than the planning CT images (shown by the blue dashed box). The position of the tooth edges in Linear_1 and Linear_2 differs greatly from the planning CT images (shown by the blue arrows). Overall, the CT value profile of sCT_Blur is closest to the planned CT, indicating that the dental tissue of sCT_Blur is more accurate compared to the other three groups of sCT.

**FIGURE 5:**
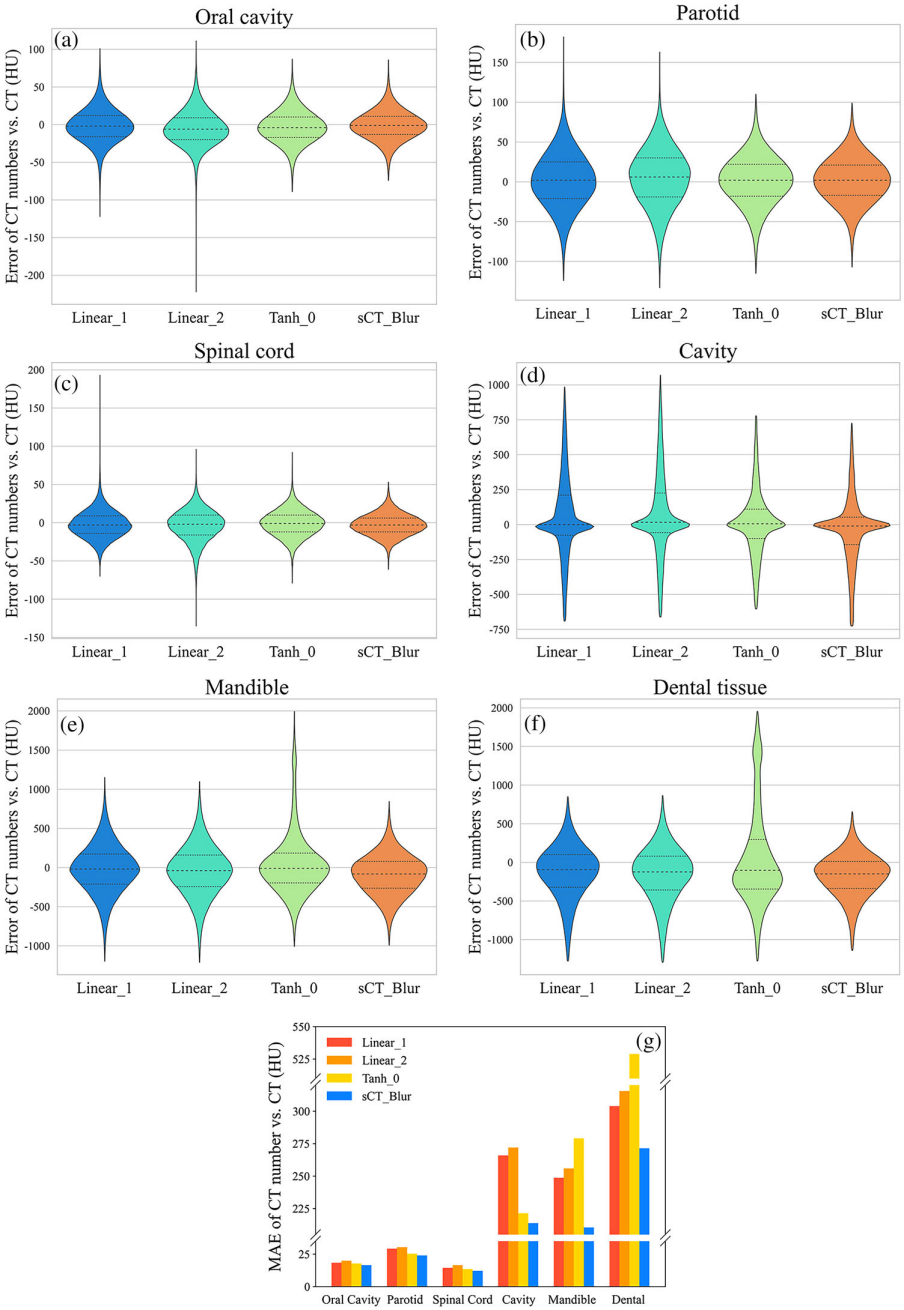
Error distributions of the CT numbers at the voxel level for (a) oral cavity, (b) parotid, (c) spinal cord, (d) cavity, (e) mandible and (f) dental tissues relative to planning CT in Linear_1, Linear_2, Tanh_0, sCT_Combine and sCT_Blur images. (g) The MAE of the CT numbers at the slice level of each OAR in the Linear_1, Linear_2, Tanh_0 and sCT_Blur images compared to the planning CT. The sCT_Blur images perform better than the other images. CT, computed tomography; MAE, mean absolute error; sCT, synthetic computed tomography. CT, computed tomography; MAE, mean absolute error; MVCBCT, megavolt cone‐beam CT.

Figure 5(a–f) show the CT number error distributions between the sCT images and planning CT images in the OARs at the voxel level, and Figure 5(g) shows the MAEs of the CT numbers for different OARs at the slice level. The CT numbers of all OARs in sCT_Blur were more accurate than those of Linear_1, Linear_2, and Tanh_0 obtained by training after normalization using conventional methods (methods I to III) in the image preprocessing phase, and the differences were statistically significant (*p* < 0.001). Relative to the planning CT images, the MAE in the CT number for each OAR in the sCT_Blur images was 15.7 ± 4.1 HU (oral cavity), 23.2 ± 7.1 HU (parotid), 11.5 ± 4.1 HU (spinal cord), 212.8 ± 104.6 HU (cavity), 219.4 ± 35.1 HU (mandible), 268.8 ± 88.8 HU (dental tissue).

## 4 DISCUSSION

**FIGURE 6:**
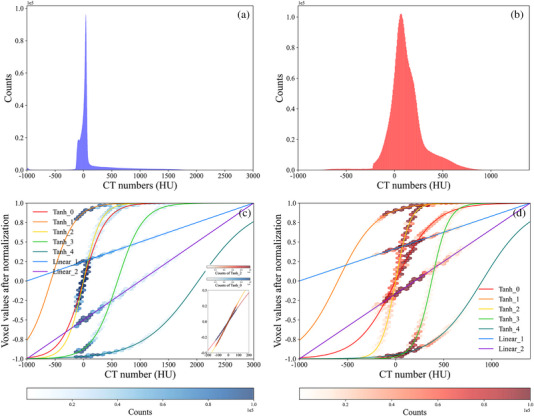
CT number histograms for the (a) CT and (b) MVCBCT images. Distributions of CT numbers and voxel values after normalization by different image preprocessing methods in the (c) CT images and (d) MVCBCT images. The depth of the hexagon color represents the number of corresponding voxel points in the images. After different image normalization preprocessing, the distribution of voxel values changes considerably. CT, computed tomography; MVCBCT, megavolt cone‐beam CT.

The main purpose of this paper is to solve the problem that the traditional, single image normalization methods (methods I to III) in the image preprocessing stage cannot allow different tissues in CT images to be adequately trained simultaneously, thus affecting the accuracy of sCT images and the accuracy of 3D dose distribution calculation in radiotherapy. We compared the accuracy of seven groups of sCT images using different normalization methods (methods I to VII) in the image preprocessing stage in different CT number intervals. We proposed a method for selecting, cropping, stitching and smoothing each group of sCT images to combine their advantages in different CT number intervals. The CT numbers in the images are widely distributed (CT: [−1000, 3000] HU, MVCBCT: [−1000, 1400] HU), and soft tissues are the main components in the images. Thus, the values of most voxels are concentrated in a small range, as shown in Figure 6(a) and (b). If the CT numbers are normalized to the range of 0 to 1 or −1 to 1 by linear normalization preprocessing methods, the values of most voxels are distributed in a smaller range (Table 3 and Figure 6c,d), resulting in poor image accuracy for the soft tissues in the sCT images (Table 4 and Figures 4 and 5). Nonlinear normalization preprocessing methods with the hyperbolic tangent function allow the voxel values of the soft tissues to fit a wider distribution after normalization (as shown in Table 3 and Figure 6c,d), thus improving the accuracy of the CT numbers of the soft tissues in the sCT images (Table 4). However, after nonlinear normalization preprocessing, the voxel values of tissues with low and high CT numbers are normalized to a very narrow range (Table 3) that could not be effectively learned. For example, after normalization preprocessing by the tanh function, the CT numbers of 500 HU, 1400 HU, and 3000 HU become 0.848, 0.998, and 0.999, respectively. As a result, the model cannot identify bones and teeth effectively. In this study, we change the distribution of voxel values in different CT number intervals after normalization by changing $a_{img}$ and $b_{img}$ in the nonlinear normalization equation (Tanh, methods III to VII) in the image preprocessing stage, thus making the CycleGAN model more focused on training image regions in specific CT number intervals. As shown in Table 3 and Figure 6(c,d), the normalization preprocessing methods used to generate the Tanh_1, Tanh_2 and Tanh_4 images allow the cavity, soft tissue and bony tissue to fit large distribution ranges, respectively. Thus, the Tanh_1, Tanh_2, and Tanh_4 images showed the best image effects in the cavity, soft tissue and bony tissue, respectively (Table 4).

The combination of multiple sCT images utilizes the advantages of various nonlinear normalization preprocessing methods to improve the accuracy of all tissues in the sCT images. Our results show that sCT_Blur image accuracy is better than that of the Tanh, Linear 1 and Linear_2 images (Tables 4 and 5). For the OARs investigated in this study, the accuracy of the CT numbers in the sCT_Blur images was higher than that in all the sCT images obtained via conventional normalization preprocessing methods; and the differences were all statistically significant (*p* < 0.001).

In a previous study, Liang et al.^24^ generated sCT images based on head and neck kVCBCT images using the CycleGAN model by applying a normalization preprocessing method involving the hyperbolic tangent function. Vinas et al.^10^ generated sCT images based on head and neck MVCBCT images using the CycleGAN model with an unknown image normalization preprocessing method. In this study, the SSIM values inside the body were recalculated according to the image quality evaluation methods proposed in the above two studies. The SSIM value is 0.928±0.014 in our study, which is higher than the results of Liang et al. (0.85±0.03) and Vinas et al. (0.927±0.028). According to the classification of soft tissues ([−400, 150] HU) and bony tissues (>150 HU) by Vinas et al.,^10^ the best MAE values of our study and Vinas’ study are 67.7 ± 11.6 HU/109.1 ± 26.2 HU (soft and bony tissues), 36.1 ± 5.9 HU/77.9 ± 17.1 HU (soft tissues), and 203.7 ± 44.4 HU/292.2 ± 69.4 HU (bony tissues), respectively. The best PSNR values of our study and Vinas' study are 29.5 ± 1.6 dB/29.7 ± 2.7 dB (soft and bony tissues), 34.9 ± 2.0 dB/31.6 ± 3.1 (soft tissues) and 23.5 ± 1.8 dB/24.8 ± 2.3 dB (bony tissues), respectively. Our results are competitive with those of Vinas et al. Moreover, in terms of the CT number errors in the OARs, the CT number errors of our results and Vinas’ results are 15.7±4.1 HU/144.0±70.6 HU (oral cavity), 23.2±7.1 HU/34.4±10.7 HU (parotid), 11.5±4.1 HU/55.5±28.1 HU (spinal cord), and 219.4±35.1 HU/233.6±61.6 HU (mandible), which proves that our results are more accurate than those in previous studies.

It is worth mentioning that the MVCBCT_2 images calibrated by the CT number‐RED calibration curve perform well in the range of [−1000, −400] HU and [150, 3000] HU but not in the soft tissue range ([−400, 150] HU), as shown in Tables 4 and 5, Figure 2 and Figure S3. Our results show that simply calibrating the MVCBCT images with CT number‐RED calibration curves is not accurate in most soft tissue regions. This problem may occur because the CT numbers in the center of the MVCBCT images are underestimated during the reconstruction process, leading to cup artifacts in the image centers. The existence of cup artifacts leads to nonuniformity in the MVCBCT images.^12^ Therefore, the calibration error is large when the CT number‐RED calibration curve obtained from the standard electron density phantom at the center of the MVCBCT beam field is applied directly before the nonuniformity is corrected. Morin et al.^12^ showed that uniformity calibration of MVCBCT images can correct cup artifacts and greatly improve the CT number accuracy in MVCBCT images. To some extent, the accuracy of the generated sCT images can be improved by training the model on CT and MVCBCT images with the artifacts removed. We will study this issue more in future work.

Our proposed method still has some limitations. In our study, we need to train the seven data sets obtained from each of the seven normalization preprocessing methods with the CycleGAN model to compare the effects of different methods on the accuracy of different tissues in the finally obtained sCT images. Obviously, this process is very time‐consuming. In addition, we cannot ensure that each normalization method is optimal because the different normalization methods are obtained by artificially changing the parameters in the normalization equation. Since the seven sets of sCT images are trained independently, the final combined sCT can be obtained only after the training of each data set is completed and cannot be integrated into the CycleGAN model for automatic training. In future work, we plan to modify the CycleGAN model to achieve automatic optimization of multiple normalization preprocesses, simultaneous training of different data obtained after normalization, and automatic combination and optimization of different sCT images.

## 5 CONCLUSION

In this study, the CycleGAN model was used to generate synthetic CT images from head and neck MVCBCT images containing dental tissue. We demonstrate that changing the image normalization preprocessing method can adjust the accuracy of different tissues in sCT, and that a single image normalization preprocessing method cannot make all tissues sufficiently accurate. Our proposed method of combining sCT can take advantage of different normalization preprocessing methods to improve the accuracy of all tissues and all OARs in sCT images, which has considerable clinical value for improving the accuracy of dose calculation in ART based on MVCBCT images.

## AUTHOR CONTRIBUTIONS

Zheng Cao conceived the experiments. Zheng Cao and Xiang Gao acquired and analyzed the data for the work. Zheng Cao, Xiang Gao, Gongfa Liu and Yuanji Pei designed the study and analyzed the result. Zheng Cao, Xiang Gao, Yankui Chang, Gongfa Liu and Yuanji Pei participated in writing manuscript. The final version of the manuscript has been reviewed and approved for publication by all authors.

## CONFLICT OF INTEREST STATEMENT

None.

## ETHICS STATEMENT

The studies were reviewed and approved by ethics committee of the First People's Hospital of Hefei (2022‐037).

## Supporting information


**Supplementary Figure 1**. Comparison of soft tissues in MVCBCT_1, CT and sCT images of different patients. The display window is [−260, 340] HU. The soft tissue images in sCT_Blur of all patients are close to the planning CT, while the anatomical structures are all consistent with MVCBCT_1. The trained model has a good generalization ability.

**Supplementary Figure 2**. Comparison of bony tissues in MVCBCT_1, CT and sCT images of different patients. The display window is [500, 2500] HU. The bony tissue images in sCT_Blur of all patients are close to the planning CT.

**Supplementary Figure 3**. Error distributions of the CT numbers in the (A) oral cavity, (B) parotid, (C) spinal cord, (D) cavity, (E) mandible and (F) dental tissues at the voxel level for the MVCBCT and sCT images. The MVCBCT images are quite different from the CT images.

## Data Availability

The original contributions presented in the study are included in the article/Supplementary Material. Further inquiries can be directed to the corresponding authors.
