# Supplementary figures and images for: Improving synthetic CT accuracy by combining the benefits of multiple normalized preprocesses
Source: J Appl Clin Med Phys. 2023 Apr 24;24(8):e14004. doi: 10.1002/acm2.14004 (PMC10402686; doi:10.1002/acm2.14004)

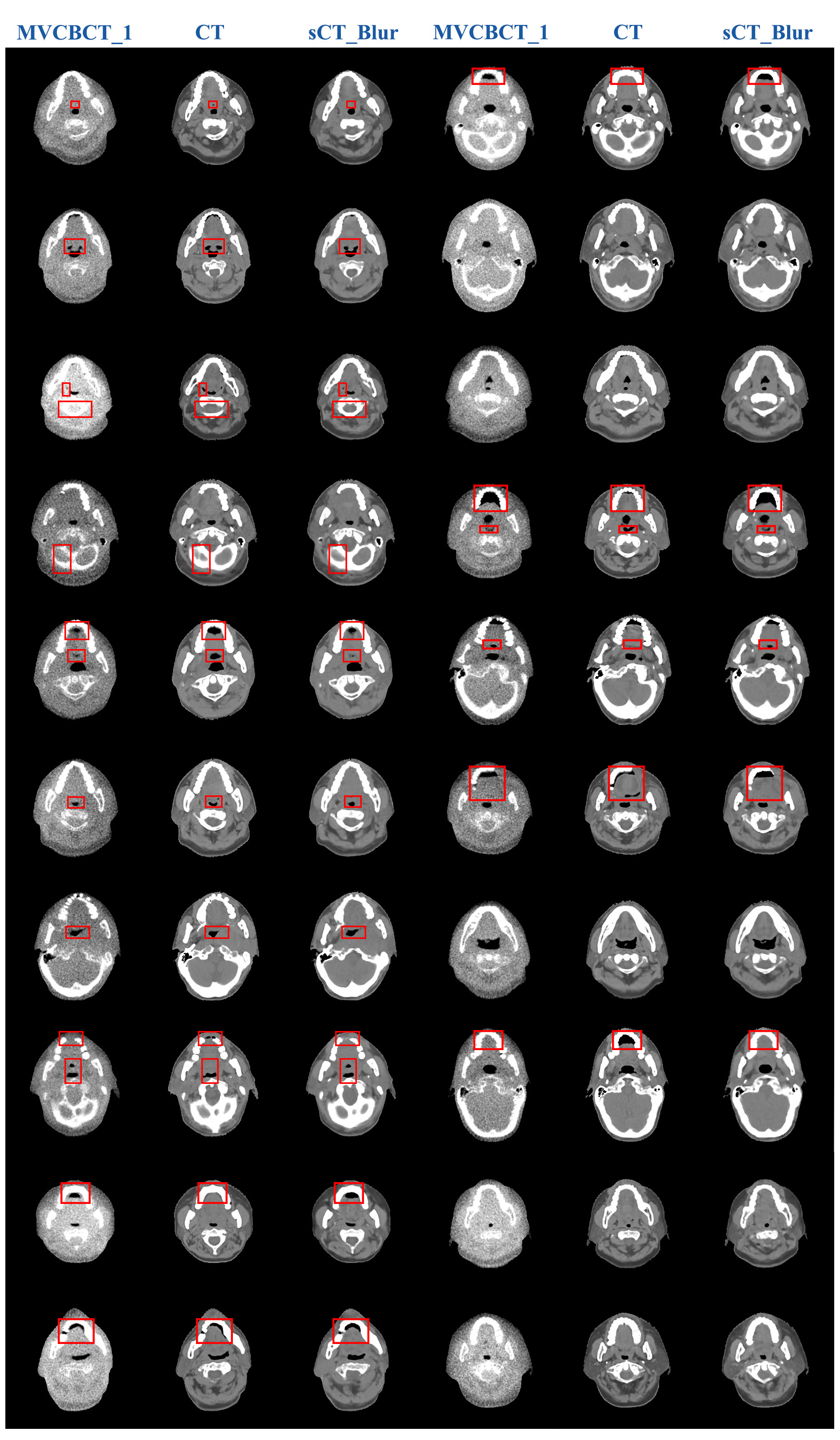

Supplement: Supplementary file 1 — Supplementary Figure 1. Comparison of soft tissues in MVCBCT_1, CT and sCT images of different patients. The display window is [−260, 340] HU. The soft tissue images in sCT_Blur of all patients are close to the planning CT, while the anatomical structures are all consistent with MVCBCT_1. The trained model has a good generalization ability. [file ACM2-24-e14004-s003.jpg]

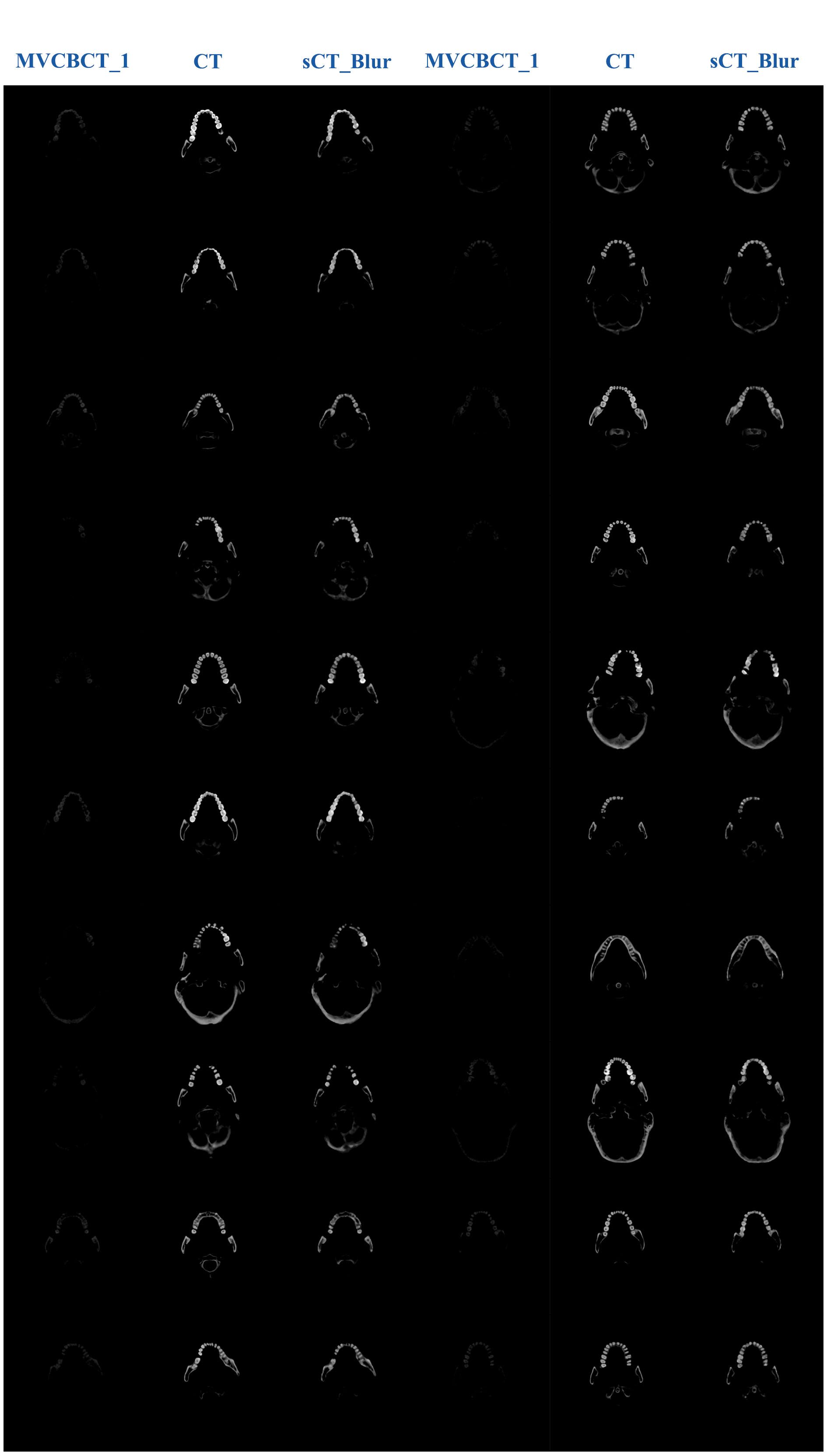

Supplement: Supplementary file 2 — Supplementary Figure 2. Comparison of bony tissues in MVCBCT_1, CT and sCT images of different patients. The display window is [500, 2500] HU. The bony tissue images in sCT_Blur of all patients are close to the planning CT. [file ACM2-24-e14004-s002.jpg]

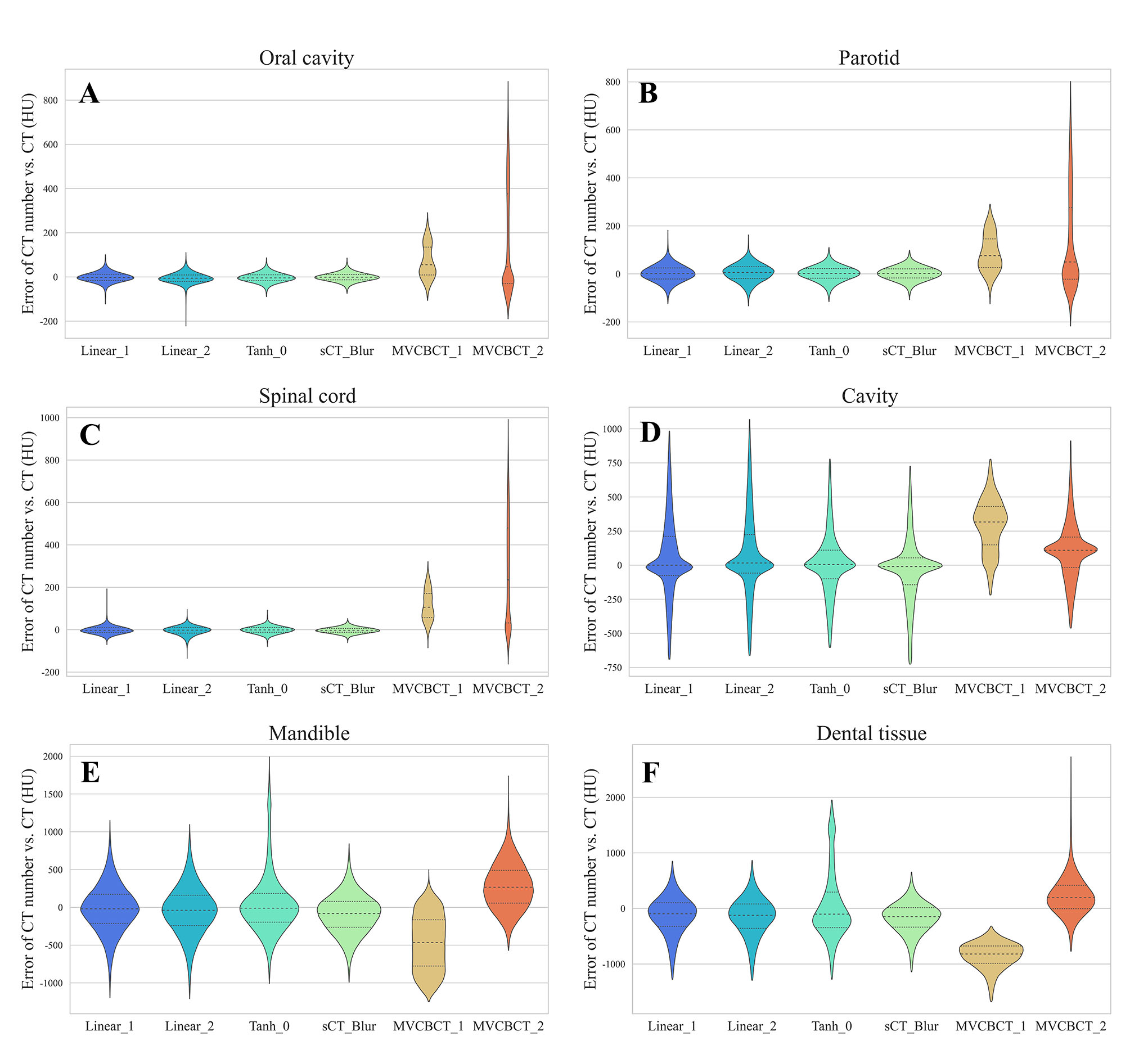

Supplement: Supplementary file 3 — Supplementary Figure 3. Error distributions of the CT numbers in the (A) oral cavity, (B) parotid, (C) spinal cord, (D) cavity, (E) mandible and (F) dental tissues at the voxel level for the MVCBCT and sCT images. The MVCBCT images are quite different from the CT images. [file ACM2-24-e14004-s001.jpg]
